# Vsx1 Transiently Defines an Early Intermediate V2 Interneuron Precursor Compartment in the Mouse Developing Spinal Cord

**DOI:** 10.3389/fnmol.2016.00145

**Published:** 2016-12-26

**Authors:** Cédric Francius, María Hidalgo-Figueroa, Stéphanie Debrulle, Barbara Pelosi, Vincent Rucchin, Kara Ronellenfitch, Elena Panayiotou, Neoklis Makrides, Kamana Misra, Audrey Harris, Hessameh Hassani, Olivier Schakman, Carlos Parras, Mengqing Xiang, Stavros Malas, Robert L. Chow, Frédéric Clotman

**Affiliations:** ^1^Laboratory of Neural Differentiation, Institute of Neuroscience, Université catholique de LouvainBrussels, Belgium; ^2^Department of Biology, University of VictoriaVictoria, BC, Canada; ^3^The Cyprus Institute of Neurology and GeneticsNicosia, Cyprus; ^4^Center for Advanced Biotechnology and Medicine and Department of Pediatrics, Rutgers University - Robert Wood Johnson Medical SchoolPiscataway, NJ, USA; ^5^Inserm U 1127, CNRS UMR 7225, Sorbonne Universités, UPMC University Paris 06 UMR S 1127, Institut du Cerveau et de la Moelle épinière (ICM)Paris, France; ^6^Laboratory of Cell Physiology, Institute of Neuroscience, Université catholique de LouvainBrussels, Belgium; ^7^State Key Laboratory of Ophthalmology, Zhongshan Ophthalmic Center, Sun Yat-sen UniversityGuangzhou, China

**Keywords:** Vsx1, V2 interneurons, spinal cord, embryonic development, neural differentiation, spinal locomotor circuits

## Abstract

Spinal ventral interneurons regulate the activity of motor neurons, thereby controlling motor activities. Interneurons arise during embryonic development from distinct progenitor domains distributed orderly along the dorso-ventral axis of the neural tube. A single ventral progenitor population named p2 generates at least five V2 interneuron subsets. Whether the diversification of V2 precursors into multiple subsets occurs within the p2 progenitor domain or involves a later compartment of early-born V2 interneurons remains unsolved. Here, we provide evidence that the p2 domain produces an intermediate V2 precursor compartment characterized by the transient expression of the transcriptional repressor *Vsx1*. These cells display an original repertoire of cellular markers distinct from that of any V2 interneuron population. They have exited the cell cycle but have not initiated neuronal differentiation. They coexpress *Vsx1* and *Foxn4*, suggesting that they can generate the known V2 interneuron populations as well as possible additional V2 subsets. Unlike V2 interneurons, the generation of Vsx1-positive precursors does not depend on the Notch signaling pathway but expression of *Vsx1* in these cells requires Pax6. Hence, the p2 progenitor domain generates an intermediate V2 precursor compartment, characterized by the presence of the transcriptional repressor Vsx1, that contributes to V2 interneuron development.

## Introduction

During CNS development, the generation of numerous neuronal populations distinct in their functional properties, location and connectivity is a prerequisite for building circuitries that regulate complex behaviors like locomotion. In vertebrates, locomotor activity is controlled by multiple regions of the CNS but is eventually triggered by local neuronal circuits that are established in the ventral spinal cord. These circuits are composed of motor neurons and of several types of interneurons that regulate motor neuron activity (Goulding, 2009; Grillner and Jessell, 2009; Garcia-Campmany et al., 2010; Grossmann et al., 2010; Lu et al., 2015). Although cardinal populations of ventral interneurons have been thoroughly described, the molecular mechanisms that regulate the generation and the diversification of these cells remain only partly elucidated.

Upon activation of unique combinations of transcription factors in response to morphogen gradients, distinct ventral interneuron progenitor domains called p0 to p3 are defined in the developing spinal cord. These progenitor domains generate four cardinal classes of ventral interneurons termed V0 to V3 (Grillner and Jessell, 2009; Garcia-Campmany et al., 2010; Lu et al., 2015). Interestingly, these cardinal populations further diversify into numerous subsets characterized by distinct combinations of molecular markers, use of different neurotransmitters, specific projection patterns and differential contributions to the locomotor circuits (Moran-Rivard et al., 2001; Pierani et al., 2001; Karunaratne et al., 2002; Sapir et al., 2004; Al-Mosawie et al., 2007; Crone et al., 2008; Zhang et al., 2008; Zagoraiou et al., 2009; Panayi et al., 2010; Dougherty et al., 2013; Francius et al., 2013; Panayiotou et al., 2013; Azim et al., 2014; Zhang et al., 2014; Britz et al., 2015). For example, V2 interneurons eventually subdivide into at least five different subtypes including V2a, V2b, V2c, V2d and Pax6^+^V2 interneurons (Zhou et al., 2000; Karunaratne et al., 2002; Al-Mosawie et al., 2007; Panayi et al., 2010; Dougherty et al., 2013; Panayiotou et al., 2013). This diversification process enables ventral interneuron subpopulations to ensure different aspects of the stereotypic pattern of locomotor activity, including left-right alternation (V0 + V2a), flexor-extensor alternation (V1 + V2b), speed (V1) and robustness and rhythmicity (V2d + V3; Lanuza et al., 2004; Gosgnach et al., 2006; Crone et al., 2008; Zhang et al., 2008, 2014; Dougherty et al., 2013; Talpalar et al., 2013; Azim et al., 2014; Britz et al., 2015).

Within the p2 domain, segregation of the V2a vs. V2b lineages depends on differential activation of the Dll4/Notch pathway (Del Barrio et al., 2007; Peng et al., 2007; Joshi et al., 2009; Skaggs et al., 2011), which relies on the mosaic expression of several transcriptional regulators including Foxn4 and Ascl1 (Li et al., 2005; Del Barrio et al., 2007; Misra et al., 2014). However, it remains unclear whether activation of the Notch pathway, V2a/V2b segregation and generation of the other V2 interneuron subsets occur within the p2 progenitor domain or in a later cell compartment of early-born V2 interneurons. In the present study, we identify an early intermediate V2 precursor compartment characterized by the transient expression of the transcription factor *Vsx1*. Vsx1 is a transcriptional repressor of the Paired-like CVC (Prd-L:CVC) homeobox gene family (Chow et al., 2001; Ohtoshi et al., 2001). In the mouse, it is expressed in gastrula stage embryos (Ohtoshi et al., 2001) and in several bipolar cone interneurons of the retina where it regulates different aspects of their terminal differentiation (Chow et al., 2001, 2004; Ohtoshi et al., 2001, 2004; Kerschensteiner et al., 2008; Shi et al., 2011, 2012). Here we show that, in the mouse developing spinal cord, Vsx1-positive cells are transiently detected from e9.5 onward, are molecularly distinct from any V2 interneuron populations and generate all the V2 populations and possibly additional V2 subsets that remain to be characterized. Loss-of-function analyses indicate that Pax6 is required for the expression of *Vsx1* in this cell compartment.

## Materials and Methods

### Mouse Strains

All experiments were performed in accordance with the European Community Council directive of 24 November 1986 (86-609/ECC) and the decree of 20 October 1987 (87-848/EEC). Mice were treated according to the principles of laboratory animal care, and experiments and mouse housing were approved by the Animal Welfare Committee of the Université catholique de Louvain (Permit Number: 2013/UCL/MD/11). The day of vaginal plug was considered as embryonic day (e)0.5. Minimum numbers of three embryos of the same genotype were analyzed in each experiment. The *Ascl1*^+/−^, *Hnf6*^+/−^; onecut 2(*Oc2*)^+/−^, Presenilin-1 (*PS1*)^+/−^, *Pax6*^+/*Sey*^ and *Vsx1^+/τLacZ^* mutant mice were previously described (Hill et al., 1992; Guillemot et al., 1993; Wong et al., 1997; Jacquemin et al., 2000; Gong et al., 2003; Chow et al., 2004; Li et al., 2004; Clotman et al., 2005). Although β-galactosidase production was evident in a ventral population in *Vsx1^τLacZ/τLacZ^* spinal cords, it was barely detectable in *Vsx1^+/τLacZ^* heterozygous embryos, probably due to the negative auto-regulatory loop reported to control *Vsx1* expression levels in the retina (Chow et al., 2004). Furthermore, β-galactosidase distribution was diffuse and punctuated, hindering the identification of the cells wherein it was present (data not shown). Therefore, a novel *Vsx1^+/nlsLacZ^* line was generated using the PG00233_Z_5_A10 allele developed by the Knock-Out Mouse Project (KOMP). *Vsx1* inactivation was confirmed by genotyping PCR and by complete loss of the Vsx1 protein. However, β-galactosidase was never detected in this line (data not shown). Nevertheless, *Vsx1^nlsLacZ/nlsLacZ^* embryos were analyzed for the development of V2 interneuron populations.

### Immunofluorescence Labelings

Mouse embryos were fixed in PBS/4% PFA at 4°C for 15–30 min according to the developmental stage. Fixed mouse embryos were washed in PBS before incubation in PBS/30% sucrose overnight at 4°C. They were embedded in PBS/7.5% gelatin/15% sucrose and frozen at −55°C. Embryos were cut at 14 μm in a Leica CM3050 cryostat.

Cryosections were saturated with PBS/0.1% Triton/10% horse serum for 30 min and incubated with the primary antibodies diluted in the same solution at 4°C overnight. For Vsx1 labeling, cryosections were permeabilized with PBS/1% Triton for 30 min at room temperature and saturated for 30 min with PBS/0.1% Triton/1% horse serum. Anti-Vsx1 antibody diluted in the same solution was incubated for 2 h at room temperature. After three washes in PBS/0.1% Triton, the secondary antibodies, diluted in PBS/0.1% Triton/10% horse serum, were added for 30 min at room temperature. Slides were washed three times in PBS/0.1% Triton before a final wash in PBS/DAPI, and were mounted with Fluorescent mounting medium (DAKO).

The following primary antibodies and dilution were used: mouse anti-Ascl1 at 1:200 (BD #556604), guinea-pig anti-Ascl1 at 1:10,000 (Kim et al., 2008), mouse anti-beta III tubulin at 1:5000 (Chemicon #MAB1637), goat anti-BhlhB5 at 1:1000 (Santa Cruz #sc-6045), rabbit or rat anti-BhlhB5 at 1:2000 (Ross et al., 2012), sheep anti-Chx10 (Vsx2) at 1:500 (Exalpha Biologicals #X1179P), goat anti Dll4 at 1:200 (R&D System #AF1389), rabbit or guinea pig anti-Foxd3 at 1:5000 (Müller et al., 2005), rat anti-Gata3 at 1: 20 (Panayi et al., 2010), guinea-pig anti-Insm1 at 1:10,000 (Welcker et al., 2013), mouse anti-Islet 1/2 at 1:6000 (DSHB #39.4D5), mouse anti-Lhx3 at 1:1000 (DSHB #67.4E12), mouse anti-Lhx1/5 at 1:2000 (DSHB # 4F2), rat anti-Nkx6.1 at 1:2 (Ono et al., 2007), guinea pig anti-OC-1 at 1:6000 (Espana and Clotman, 2012), sheep anti-OC-1 at 1:250 (R&D System #AF6277), rat anti-OC-2 at 1:400 (Clotman et al., 2005), mouse anti-p27^kip1^ at 1:2000 (BD), mouse anti-Pax6 at 1:1000 (DSHB #PAX6), mouse anti-phospho-Histone H3 at 1:1000 (Abcam ab14955), rabbit or guinea pig anti-Prdm8 at 1:1000 (Ross et al., 2012), goat anti-Prox1 at 1:100 (R&D Systems # AF2727), mouse anti-Shox2 at 1:500 (Abcam #ab55740), goat anti-Sox 1 at 1:500 (Santa Cruz #sc-17318), rabbit anti-Vsx1 at 1:500 (Clark et al., 2008).

Secondary antibodies from Life Science were used at 1:2000 and were donkey anti-mouse/AlexaFluor 594 or 488 or 647, anti-rabbit/AlexaFluor 647, anti-rat/AlexaFluor 594, anti-goat/AlexaFluor 488, anti-sheep/AlexaFluor 594, anti-mouse IgG_1_/AlexaFluor 594 or anti-mouse IgG_2a_/AlexaFluor 488. Secondary antibodies from Jackson ImmunoResearch were used at 1:1000 and were donkey-anti chicken/Dylight 488 or 594, anti-mouse/AlexaFluor 647, anti-rat/AlexaFluor 647, anti-sheep/AlexaFluor 594 or 647, anti-rabbit/AlexaFluor 488 or 594, anti-guinea pig/AlexaFluor 488 or 647. All secondary antibodies gave signals in the spinal cord only in the presence of corresponding primary antibodies.

### Double *in situ* Hybridization (ISH)

Mouse embryos were fixed overnight in PBS/4% PFA at 4°C and processed as for immunolabeling experiments. Fourteen micrometer cryostat sections were cut and double *in situ* hybridization (ISH) protocol was performed essentially as previously described with slight modifications (Beguin et al., 2013; Pelosi et al., 2014). Sections were simultaneously hybridized overnight at 65°C with a DIG-conjugated *Foxn4* (NM_148935.2, nucleotides 78–1643 (Francius et al., 2015)) and a fluorescein-labeled *Vsx1* (NM_054068.2, nucleotides 121–2728, provided by C. Cepko) riboprobe. After hybridization, sections were washed four times in 50% Formamide, 1× SSC, 0.1% Tween-20 for 1 h at 65°C, twice in MABT buffer (100 mM maleic acid, 150 mM NaCl, 0.1% Tween20, pH 7.5) for 30 min before blocking in blocking buffer (MABT, 2% blocking reagent from Roche, 20% inactivated horse serum) for 2 h at room temperature. Sections were then incubated overnight with anti-DIG-alkaline phosphatase (AP)-conjugate antibody (Roche) at 4°C. After washing for 30 min in MABT, *Foxn4* probe was visualized by AP-catalyzed chromogenic reaction using NBT-BCIP substrates (Roche) according to manufacturer’s instructions. The color reaction was stopped in 1× PBS and AP was inactivated by incubating the slides for 30 min with 0.1 M glycine/HCl pH 2.2. The sections were then washed twice in MABT buffer for 30 min, blocked again in blocking buffer for 2 h, and incubated to a 1:2000 dilution of anti-Fluo-AP-conjugate antibody (Roche) in blocking buffer overnight at 4°C. Slides were washed and incubated with HNPP/Fast Red kit (Roche) according to the manufacturer’s instructions to visualize *Vsx1* expression. The reaction was stopped by washing in 1× PBS. Sections were then counterstained with DAPI and slides were mounted with Fluorescent mounting medium (DAKO).

### Imaging and Quantitative Analyses

Immunofluorescence and double ISH images of cryosections at thoracic level of the developing spinal cord were acquired on a Zeiss Axio Cell Observer Z1 confocal microscope with the Zeiss AxioVision Rel. 4.8 software and processed with Adobe Photoshop CS5 software. Double ISH images were also acquired using an EVOS^®^ FL Auto Imaging System (ThermoFisher Scientific) and related software. Quantifications were performed on red or green or blue layer of acquired confocal images and double or triple labeling cells were processed by subtractive method (Francius and Clotman, 2010). For each embryo (*n* ≥ 3), both sides of three sections at thoracic level were quantified using the count analysis tool of Adobe Photoshop CS5 software.

Raw data were exported from Adobe Photoshop CS5 software to SigmaPlot v12.3 software and processed in order to generate histogram figures. All data were analyzed and histograms were made with SigmaPlot software. Adequate statistical tests were applied based on the number of comparisons and on the variance in each group. For analysis of cell quantification based on comparison of two groups (control or mutant), standard Student’s *t*-tests were performed. Quantitative analyses were considered significant at *p* < 0.05. Three asterisks (***) indicate *p* < 0.001.

## Results

### The p2 Progenitor Domain Generates an Early V2 Population

**Figure 1:**
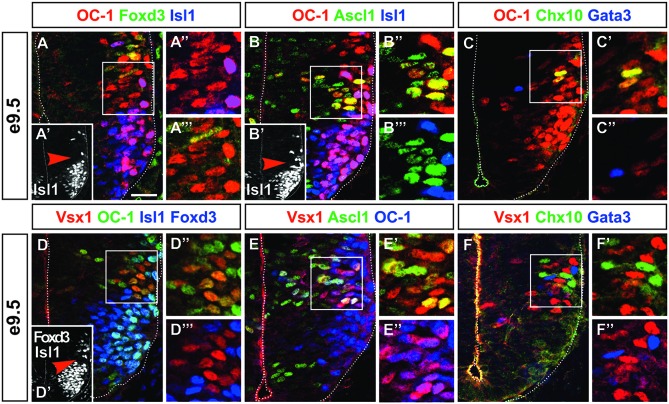
**Onecut (OC)-1 and Vsx1 are present in an early V2 interneuron population. (A–A″′)** At e9.5, OC-1 is detected in cells located between motor neurons (Isl1+, inset **A′**; arrowhead indicates the location of **A″** and **A″′** insets) and V1 interneurons (Foxd3+). **(B–B″′)** These cells derive from the p2 progenitor domain (Ascl1+) and are not motor neurons (Isl1+, inset **B′**; arrowhead indicates the location of **B″** and **B″′** insets). **(C–C″)** OC-1 is detected in the V2 domain in cells that are neither V2a (Chx10+) nor V2b (Gata3+) interneurons. **(D–D″′)** Vsx1 is detected in cells containing OC-1 located between motor neurons (Isl1+, inset **D′**; arrowhead indicates the location of **D″** and **D″′** insets) and V1 interneurons (Foxd3+). **(E–E″)** Vsx1 is present in cells that derive from the p2 domain (Ascl1+) and contain OC-1. **(F–F″)** Vsx1^+^ cells are distinct from early V2a (Chx10+) and V2b (Gata3+) interneurons. Scale bar = 50 μm.

**Figure 2:**
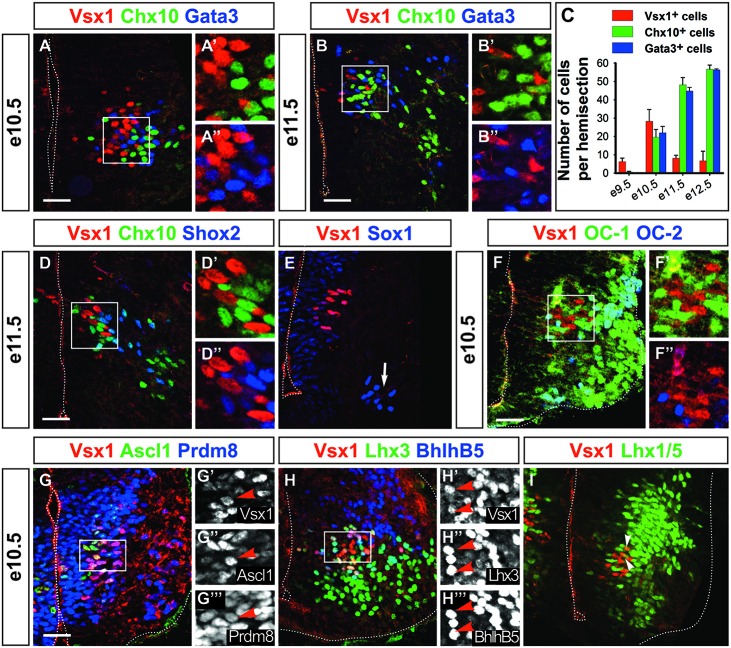
**Vsx1 identifies a population distinct from V2 interneurons.** At e10.5 **(A–A″)** and e11.5 **(B–B″)**, Vsx1 is present in cells that intermingle with, but are distinct from V2a (Chx10+) and V2b (Gata3+) interneurons. **(C)** Quantification of V2a, V2b and Vsx1^+^ cells at different stages (mean values ± SEM, *n* = 3). **(D–D″)** Vsx1 is not detected in V2d interneurons (Shox2 + Chx10−). **(E)** Vsx1^+^ cells are also distinct from V2c cells (Sox1+, arrow). **(F–F″)** At e10.5, OC-1 is present at low levels in Vsx1^+^ cells, while OC-2 is undetectable. **(G–G″′)** Ascl1 is present in Vsx1^+^ cells, confirming their p2 origin. Proteins present in V2a, including Prdm8 **(G–G″′)**, Lhx3 and Bhlhb5 **(H–H″′)**, are also detected. **(I)** In contrast, Lhx1/5, which is found in V2b, is barely detectable (arrowheads). Scale bars = 50 μm.

In mouse embryonic spinal cord, neurogenesis starts around e9.5. The earliest differentiating neurons are motor neurons, which originate from the pMN progenitor domain. We recently described that the production of V1 interneurons is also initiated at e9.5 (Stam et al., 2012). At this early stage, the presence of the OC transcription factors, namely OC-1 (or HNF-6), OC-2 and OC-3, was restricted to differentiating neurons and was observed in motor neurons and V1 interneurons (Francius and Clotman, 2010; Roy et al., 2012; Stam et al., 2012; Francius et al., 2013). Surprisingly, OC-1 was additionally detected in cells located between motor neurons and prospective Renshaw cells (Figures 1A–A″′; Stam et al., 2012). As the p2 progenitors are located between the pMN and the p1 domains, we postulated that these unidentified cells were most likely early-born V2 cells containing OC factors. To test this hypothesis we assessed the distribution of Ascl1, which is specifically detected in the p2 domain and early V2 interneurons (Gong et al., 2003; Wildner et al., 2006; Parras et al., 2007). Co-detection of OC-1 and Ascl1 confirmed that early-born OC-1^+^ cells derived from the p2 domain (Figures 1B–B″′). Given that OC factors are detected only in post-mitotic neurons (Francius and Clotman, 2010), we concluded that these cells were early V2 interneurons. However, V2 interneurons are reported to be generated from e10.5 onward (Zhou et al., 2000; Li et al., 2005; Peng et al., 2007). Accordingly, the number of V2a or V2b identified by the presence of Chx10 or Gata3, respectively, was restricted at e9.5 (1–5 cells per hemisection; Figures 1C–C″,F–F″, 2C) but significantly increased from e10.5 on (Figures 2A,C; Nardelli et al., 1999; Zhou et al., 2000; Smith et al., 2002). At e9.5, OC-1 was present in V2a and V2b interneurons, as well as in other cells located lateral to the p2 progenitor domain (Figures 1C–C″). Taken together, these observations suggested either that OC-1 was detected in early V2a/V2b interneurons before the onset of *Chx10*/*Gata3* expression or that OC-1 was present in an additional unknown V2 subset. The latter hypothesis was consistent with results from a genetic lineage-tracing of Foxn4^+^ progenitors, which indicated that the p2 domain produces additional unidentified V2 interneurons (Li et al., 2010).

To distinguish between these possibilities, we searched for specific markers of these cells. Several transcription factors present in V2 interneurons are also detected in differentiating neurons of the retina (Glaser et al., 1992; Liu et al., 1994; Tomita et al., 1996; Gouge et al., 2001). Thus, specific markers of this V2 subset may be found among retinal proteins that are not yet associated with a specific spinal population. Vsx1 is the single paralog of the V2a marker Chx10 in the mouse genome and is expressed as Chx10 in the developing retina (Chow et al., 2001; Ohtoshi et al., 2001). Interestingly, *Vsx1* expression in retinal cells is inhibited by Chx10 (Clark et al., 2008) and *Chx10* expression is repressed by Vsx1 in Type 7 cone bipolar cells (Shi et al., 2011). In the zebrafish embryonic spinal cord, the Vsx1 ortholog is detected in common V2a/V2b progenitors and is transiently maintained in the V2a lineage (Batista et al., 2008; Kimura et al., 2008). In Xenopus and chick, *Vsx1* expression was attributed to V2a interneurons based on its distribution similar to Chx10 (Chen and Cepko, 2000; D’Autilia et al., 2006). However, co-labeling of Vsx1 and other V2 markers in the mouse embryonic spinal cord was not reported yet.

Therefore, we characterized the distribution of Vsx1 in the mouse developing spinal cord. First, we assessed whether Vsx1 is present in the early OC-1^+^ V2 cells identified at e9.5. At this stage, Vsx1 was detected in cells containing OC-1 that were neither motor neurons nor early V1 interneurons (Figures 1D–D″′). As observed for OC-1 (Figures 1B–B″′), these cells contained Ascl1, confirming that they derived from the p2 domain (Figures 1E–E″). To determine whether these may correspond to V2a or V2b interneurons, the distribution of Vsx1 was compared to that of Chx10 and Gata3. However, Vsx1 was detected in cells lacking Chx10 or Gata3 (Figures 1F–F″), leaving open the question whether they corresponded to early V2 precursors or to a distinct population of V2 interneurons.

### Vsx1 Defines a Specific V2 Subset

To further investigate this question, we compared the distribution of Vsx1 and V2 markers at later developmental stages (Figures 2A–E). We first noticed that the number of Vsx1^+^ cells increased from e9.5 to e10.5, then decreased to e12.5 and became undetectable at later stages (Figures 2A–C and data not shown), indicating a transient expression of *Vsx1* in the mouse spinal cord as observed in zebrafish embryos (Passini et al., 1998). At all developmental stages, Vsx1 was found in cells located in close vicinity to V2a and V2b interneurons. However, co-detection of Vsx1 with Chx10 or Gata3 was never observed (Figures 2A–B″). Vsx1 was also present at e11.5 in cells located nearby V2d interneurons, characterized by the presence of Shox2 in the absence of Chx10 (Dougherty et al., 2013), but Shox2 was not detected in Vsx1^+^ cells (Figures 2D–D″). Finally, Vsx1 distribution was compared to that of Sox1, which labels V2c interneurons in addition to spinal progenitors (Panayi et al., 2010). However, V2c are located more ventrally and were distinct from cells containing Vsx1 (Figure 2E, arrow). Hence, Vsx1 is a specific, albeit transient, marker of a novel cell subset distinct from described V2 populations.

Spinal neurons that share lineage relationship or developmental origin often exhibit common markers. To assess whether Vsx1^+^ cells might relate to any V2 population, we established a repertoire of markers detected in these cells. As OC-1 was detected in Vsx1^+^ cells at early developmental stages (Figures 1D–D″), we assessed whether OC factors were co-detected with Vsx1 in more differentiated cells. At e10.5, OC-1 levels decreased sharply but the protein was still detected in 66% of the Vsx1^+^ cells (Figures 2F–F″), while OC-2 (Figures 2F–F″) and OC-3 (data not shown) were absent. As observed at e9.5, Ascl1 was present at e10.5 in the Vsx1^+^ cells, confirming their p2 origin (Figures 2G–G″′). Lhx3 is present in pMN and in p2 progenitors and is maintained in the medial motor column and in V2a interneurons but not in other V2 subsets. Lhx3 was detected in 95% of the Vsx1^+^ cells (Figures 2H–H″′), suggesting that they might relate to V2a interneurons. Accordingly, Prdm8 and Bhlhb5, which are present in V2a interneurons (Skaggs et al., 2011; Francius et al., 2013), were detected in 97% and 87% of the Vsx1^+^ cells, respectively (Figures 2G–H″′). In contrast Lhx1/5, which is present in V2b but is excluded from V2a interneurons, was barely detectable in 7% of the Vsx1^+^ complement (Figure 2I, arrowheads). Thus, as previously reported in other species (Chen and Cepko, 2000; D’Autilia et al., 2006; Batista et al., 2008; Kimura et al., 2008), Vsx1^+^ cells share some characteristics with V2a interneurons but Vsx1 is not detected in known V2 populations.

### Vsx1 Is Restricted to an Early V2 Compartment Prior to Neuronal Differentiation

**Figure 3:**
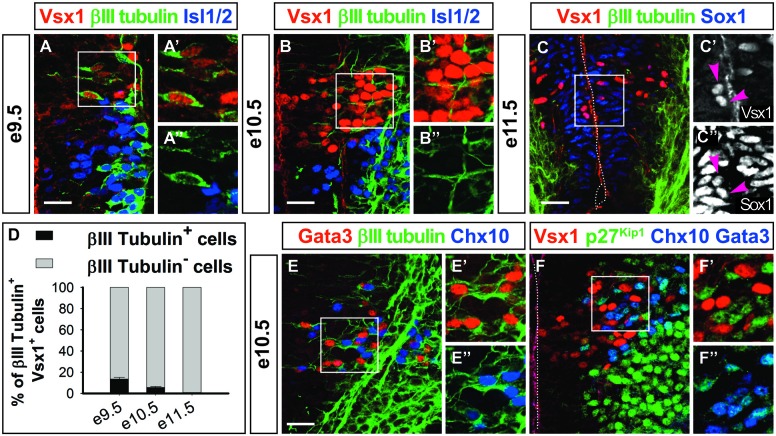
**Vsx1 is present before the onset of neuronal differentiation. (A–B″)** At e9.5 **(A–A″)** and e10.5 **(B–B″)**, the majority of the Vsx1^+^ cells are devoid of β-_III_-tubulin. In contrast, most of the motor neurons (Isl1/2+) contain β-_III_-tubulin at this stage. **(C–C″)** Co-labeling of β-_III_-tubulin and Vsx1 is never observed at e11.5, and the Vsx1^+^ cells are located between the neural progenitors (Sox1+) and the differentiating neurons (β-_III_-tubulin+). Vsx1 is detected in some Sox1+ progenitors (arrowheads). **(D)** Quantifications (mean percentage ± SD, *n* = 3) shows that only a minority of Vsx1^+^ cells contain β-_III_-tubulin at e9.5 and e10.5 and none at e11.5. **(E–E″)** In contrast, at e10.5, β-_III_-tubulin is readily detected in differentiating V2a (Chx10+) or V2b (Gata3+) interneurons. **(F–F″)** Accordingly, p27^Kip^ is present in V2a and V2b interneurons (Chx10+ and Gata3+, both in blue) but not in Vsx1^+^ cells. Scale bars = 50 μm.

The lack of differentiated V2 interneuron markers in Vsx1^+^ cells and their medial location in the intermediate zone (Figures 1, 2) prompted us to assess whether they were differentiating neurons. Co-labeling experiments from e9.5 to e11.5 showed that only a minority of the Vsx1^+^ cells, decreasing from 14% to 0%, respectively, contained the neuronal differentiation marker β-_III_-tubulin (Figures 3A–D). This suggested that Vsx1 was present before the onset of neuronal differentiation in p2-derived cells. Accordingly, Sox1, which labels the spinal progenitors in the ventricular zone, was detected in medial Vsx1^+^ cells located within the subventricular zone (Figures 2E, 3C–C″). These observations supported the hypothesis that *Vsx1* is expressed in early V2 precursors before the onset of expression of their population-specific differentiation markers. Consistent with this idea, β-_III_-tubulin was detected in all the V2a and V2b cells at e10.5 (Figures 3E–E″) and the cyclin-dependent kinase inhibitor protein p27^Kip1^ was present in V2a and V2b interneurons but not in Vsx1^+^ cells (Figures 3F–F″, Chx10 and Gata3 both in blue). This suggested that *Vsx1* expression occurs earlier than that of *Chx10* and *Gata3* in V2 interneurons. Hence, Vsx1 may be restricted to an early V2 compartment prior to V2 interneuron diversification.

**Figure 4:**
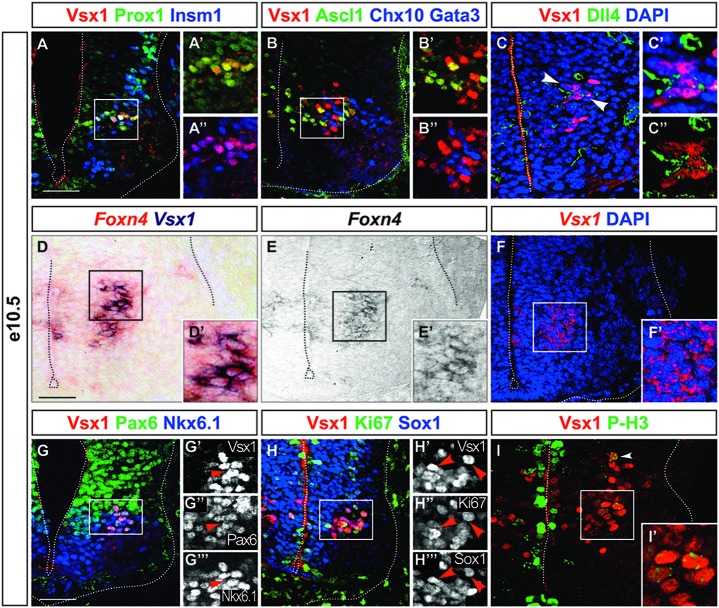
**Vsx1 is present in an early intermediate compartment during V2 interneuron differentiation. (A–A″)** Vsx1 is detected in the intermediate compartment characterized by the presence of Prox1 and Insm1. **(B–B″)** Ascl1 is co-detected with Vsx1, which is not the case for Chx10 and Gata3 (both in blue). **(C–C″)** Dll4 is detected in Vsx1^+^ cells (arrowheads). **(D–F′)** *Vsx1* mRNA is detected by double *in situ* hybridization (ISH) in cells expressing *Foxn4*. **(G–G″′)** Pax6 and Nkx6.1 are maintained in cells containing Vsx1. **(H–H″′)** Although Sox1 is present at low levels in some Vsx1^+^ cells, Ki-67 is barely detectable. **(I,I′)** Only remaining traces (arrowhead) of phospho-HistoneH3 is detected in cells containing Vsx1, as compared to the strong labeling of the dividing progenitors in the ventricular zone. Scale bars = 50 μm.

To test this idea, we compared the distribution of Vsx1 with that of Prox1 and Insm1. These two factors define an early intermediate compartment containing late progenitors (i.e., progenitors in their last division) and newborn neurons (Duggan et al., 2008; Misra et al., 2008). Indeed, we found that Vsx1 was present in cells containing both Prox1 and Insm1 (Figures 4A–A″), demonstrating that Vsx1 is restricted to an early intermediate V2 subset. The presence of Ascl1 in this subset but not in differentiating V2a or V2b interneurons (Figures 1B–B″, 2G–G″, 4B–B″, Chx10 and Gata3 both in blue) suggested that the Vsx1^+^ cells may correspond to precursors of the described V2 populations. We thus investigated whether Vsx1^+^ cells contained other determinants of V2 development. The segregation of V2a and V2b interneurons depends on a regulatory network involving Ascl1 and Foxn4 that eventually controls production of the Notch ligand Dll4 and thereby differentially modulates activation of Notch signaling in V2 precursors (Li et al., 2005; Del Barrio et al., 2007; Peng et al., 2007; Misra et al., 2014; Zou et al., 2015). Dll4 was present in Vsx1^+^ cells at the time of V2a/V2b production (Figures 4C–C″, arrowheads), although it was not exclusive to the Vsx1^+^ population. Similarly, *Foxn4* expression was detected by double ISH in the cells expressing *Vsx1* (Figures 4D–F′). As V2 precursors expressing *Foxn4* have been shown to generate all the described V2 interneuron populations as well as additional V2 subsets (Li et al., 2010), our data demonstrate that Vsx1^+^ cells are precursors of V2a/V2b interneurons and of other V2 subsets that also derive from Foxn4- and Dll4-containing progenitors (Panayi et al., 2010; Panayiotou et al., 2013; Zou et al., 2015).

We then assessed whether Vsx1 is detected in dividing cells of the ventricular zone or in intermediate precursor cells. Pax6 and Nkx6.1, which are present in the p2 progenitors, were detected in the Vsx1^+^ cells. However, these cells were clustered nearby the lateral border of the ventricular zone rather than the lumen (Figures 4G–G″′). Consistently, although Sox1 was present at low levels (Figures 2E, 4H–H″′), the proliferation marker Ki-67 was barely detectable in Vsx1^+^ cells (Figures 4H–H″′) and only remaining traces of phosphorylated-Histone H3, specific to the metaphase of mitotic division, were found (Figures 4I,I′, arrowhead). Taken together, these observations suggest that Vsx1 is present in cells that just completed their last mitotic division but did not yet initiate neuronal differentiation, defining an early intermediate V2 interneuron compartment. As observed respectively in motor neurons and in V1 interneurons, Nkx6.1 and Pax6 are maintained in these cells even though they are no longer dividing.

### The Developmental Determinants of V2 Interneurons Are not Required for the Production of Vsx1^+^ Cells

**Figure 5:**
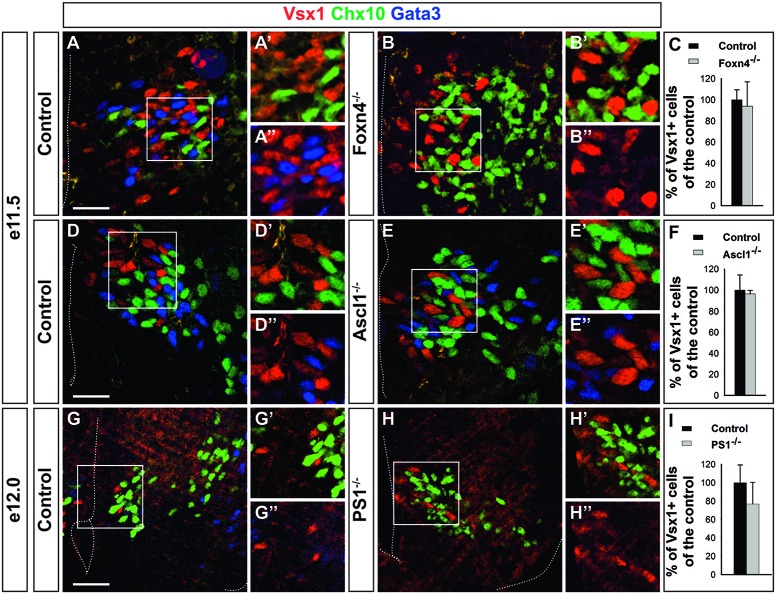
**The generation of Vsx1^+^ cells does not require Notch signaling. (A–C)** The generation of Vsx1^+^ cells is not altered in *Foxn4* mutant embryos, although production of V2b interneurons (Gata3+) is abolished in favor of V2a interneurons (Chx10+). **(D–F)** Vsx1^+^ cell production is not altered in *Ascl1* mutant embryos whereas supernumerary V2a interneurons are generated at the expense of V2b cells. **(G–I)** In *Presenilin-1* (*PS1*) mutant embryos, the number of Vsx1^+^ cells was similar to that of control embryos whereas V2b interneurons were replaced by V2a interneurons. Scale bars = 50 μm. Quantifications are expressed as mean percentage ± SD, *n* = 4.

Given the possibility that Vsx1^+^ cells may constitute precursors of V2 populations, we assessed whether their production depends on factors necessary for proper V2 interneuron development. The segregation of the V2a and V2b lineages relies on the asymmetrical activation of the Notch pathway by Dll4, which stimulates Notch signaling in surrounding cells and thereby promotes V2b differentiation, restricting V2a differentiation to cells with lower Notch activity (Li et al., 2005; Del Barrio et al., 2007; Peng et al., 2007; Misra et al., 2014; Zou et al., 2015). Proper activation of this pathway requires mosaic expression of several transcriptional regulators including Foxn4 and Ascl1 in the p2 domain (Li et al., 2005; Del Barrio et al., 2007; Xiang and Li, 2013; Misra et al., 2014). To assess whether the production of the Vsx1^+^ cells also depends on this network, we evaluated the phenotype of these populations in embryos mutant for *Foxn4*, *Ascl1* or *PS1*. Lack of *Foxn4* resulted as expected in a strong imbalance in the generation of V2 interneuron subsets, with V2a being produced in excess at the expense of V2b cells (Figures 5A–B″). However, the generation of Vsx1^+^ cells was not affected (Figures 5A–C). Similarly, inactivation of *Ascl1* resulted in excessive production of V2a interneurons at the expense of V2b, but the number of Vsx1^+^ cells was not altered (Figures 5D–F). Consistently, loss of PS1, which results in severe diminution of Notch activation (Struhl and Greenwald, 2001), did not alter the specification of Vsx1^+^ cells (Figures 5G–I). These observations indicate that the generation of Vsx1^+^ cells does not depend on Notch signaling, but remain consistent with the hypothesis that these cells may constitute the precursor population wherein differential activation of the Notch pathway determines further V2 interneuron diversification.

### Pax6 Is Required for Proper Expression of *Vsx1* in V2 Precursors

**Figure 6:**
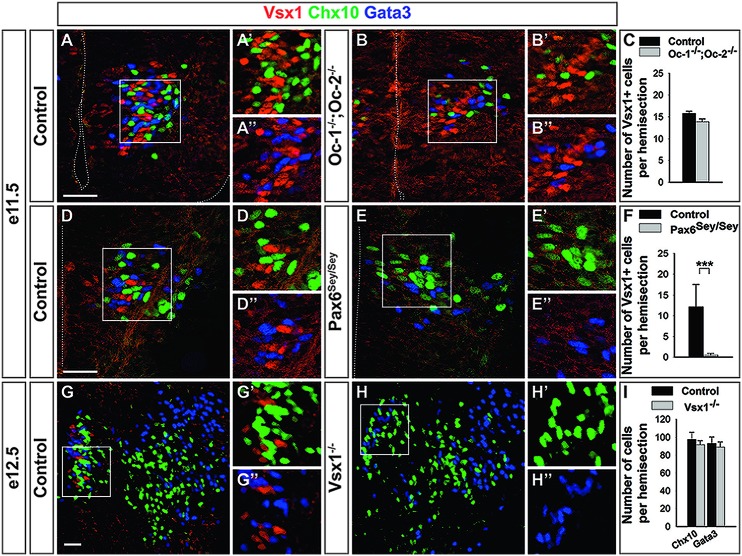
**Pax6 is necessary for Vsx1 expression. (A–C)** The generation of Vsx1^+^, Chx10 + and Gata3 + cells, is not affected in *Oc1^−/−^Oc2^−/−^* embryos. **(D–F)** In *Pax6^Sey/Sey^* embryos, the number of Vsx1^+^ cells is dramatically reduced (***p < 0.001) without similar alteration of V2a or V2b production. **(G–I)** In embryos lacking Vsx1, the production of V2a and of V2b interneurons is unaffected. Scale bars = 50 μm. Quantifications are expressed as mean values ± SEM, *n* = 3.

We then turned our analyses to the other transcription factors expressed during Vsx1^+^ cell development. OC factors are present in the early Vsx1^+^ cells (Figures 1, 2). The role of OC factors has been evaluated in *Oc1/Oc2^−/−^* double-mutant embryos, which also lack Oc3 in the CNS (Roy et al., 2012). However, the number of Vsx1^+^ cells was not changed in the spinal cord of these mutants, suggesting that OC proteins are not necessary for their production (Figures 6A–C).

Next, we investigated the role of Pax6 in Vsx1^+^ cell generation. Pax6 is required for patterning the ventral spinal cord during neurogenesis (Ericson et al., 1997) and for proper generation of V1 interneurons (Burrill et al., 1997; Sapir et al., 2004; Gosgnach et al., 2006). In *Small eye* (*Sey/Sey*) mutant embryos, which lack Pax6 (Glaser et al., 1992), the production of V0 and V2b populations is not affected (Burrill et al., 1997; Sapir et al., 2004; Gosgnach et al., 2006; Zhang et al., 2014) while the number of V2a interneurons is only slightly decreased (Ericson et al., 1997; Gosgnach et al., 2006). The persistence of Pax6 in the early Vsx1^+^ cells suggested that this factor may contribute to their development. Accordingly, Vsx1 was barely detectable in *Sey/Sey* embryos at e11.5 (Figures 6D–F) and e12.5 (data not shown), although the production of V2a and V2b interneurons was conserved. The presence of V2a and V2b interneurons indicated that the p2 progenitor domain was not re-specified and that the V2a/V2b precursors were preserved. In contrast, the loss of Vsx1 unveiled a selective requirement for Pax6 in the expression of Vsx1 in this early intermediate V2 compartment. Furthermore, these observations suggest that Vsx1 may be dispensable for proper generation of V2 interneuron subsets.

To test this hypothesis, we assessed the role of Vsx1 in V2 interneuron development. In the absence of any other specific marker of the Vsx1^+^ cells, this compartment could not be analyzed in the *Vsx1* mutant embryos. In contrast, as Vsx1-containing precursors generate all the V2 subsets, we addressed a possible role for Vsx1 in V2 interneuron development. An inverse relationship between the levels of Chx10 and Vsx1 was previously reported in bipolar cells of the retina (Clark et al., 2008). To investigate whether a similar relationship also exists in spinal cord, the distribution of Chx10 was first assessed in *Vsx1* mutant embryos. However, the number of Chx10-positive cells was similar at e12.5 in control and in mutant embryos, indicating that the absence of Vsx1 does neither impact on V2a interneuron production nor on *Chx10* expression. Accordingly, the number of V2b interneurons was not affected and the V2a/V2b ratio was preserved (Figures 6G–I). Taken together, these observations confirm that Vsx1 is not required for the production of described V2 interneuron populations, suggesting that the integrity of the early V2 precursor compartment is also preserved in *Vsx1* mutant embryos. Consistently, *Vsx1* mutant mice did not display any alteration in their motor behavior (data not shown).

## Discussion

**Figure 7:**
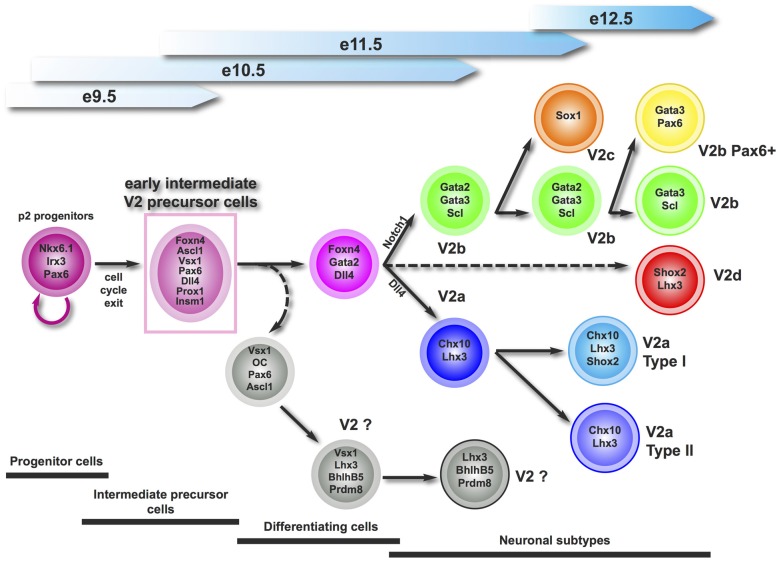
**Proposed working model for the generation of V2 interneuron subtypes.** Vsx1 defines an early intermediate precursor compartment that generates all the described V2 interneuron subsets, and may produce additional V2 subpopulation(s) that remain(s) to be characterized.

During spinal cord development, ventral progenitor domains produce multiple neuronal populations that variously contribute to the activity of motor circuits. Here, we provide evidence that the p2 progenitor domain generates an early intermediate compartment characterized by the presence of Vsx1 prior to neuronal differentiation. This early compartment very likely produces the collection of known V2 interneuron populations, and may also generate additional V2 subsets that remain to be identified (Figure 7).

In the zebrafish embryonic spinal cord, Vsx1 is detected in common V2a/V2b progenitors and its expression is transiently maintained in the V2a lineage (Batista et al., 2008; Kimura et al., 2008). In Xenopus and chick, the expression of *Vsx1* was attributed to V2a interneurons based on its distribution similar to that of Chx10 (Chen and Cepko, 2000; D’Autilia et al., 2006). Here, we demonstrate that, in contrast to other species, murine Vsx1 is restricted to an early intermediate compartment that likely comprises precursors of the known V2 subsets. Although they contain factors known as progenitor markers, including Sox1, Nkx6.1 and Pax6, Vsx1^+^ cells are not proliferating as evidenced by the absence of proliferation or mitosis markers. Conversely, Vsx1^+^ cells are not differentiating neurons, as they neither display cyclin-dependant kinase inhibitor protein of V2 interneurons (Gui et al., 2007) and very few of them produce β-_III_-tubulin. Therefore, they constitute an early intermediate compartment of V2 postmitotic precursors (Figure 7).

Consistent with this idea, Vsx1^+^ cells contain Prox1 and Insm1, which specifically label compartments comprising late progenitors and newborn neurons and are transiently expressed before the onset of neuronal differentiation (Duggan et al., 2008; Misra et al., 2008). Prox1 acts downstream of the pro-neural proteins Ascl1 and Neurog-2 to implement neurogenesis in the spinal interneurons and may antagonize the anti-neurogenic activity of Sox proteins (Misra et al., 2008) including Sox1, which is present in spinal progenitors. Insm1 is thought to play a role in the termination of progenitor proliferation throughout the nervous system (Duggan et al., 2008). Prox1 and Insm1 may affect the onset and extent of terminal and penultimate neurogenic divisions, and thus the total number of neurons produced, in any area and stage of neurogenesis. In the spinal intermediate V2 compartment defined by the presence of Vsx1, one may additionally propose that Prox1 and Insm1 could delay the onset of neuronal differentiation and thereby open a time-window necessary for the activation of signaling pathways, including Notch, that will determine the diversification of V2 precursors in multiple V2 populations.

Our data suggest that Vsx1^+^ cells are the precursors of most of the V2 interneurons (Figure 7) including V2a and V2b subsets (Li et al., 2005; Del Barrio et al., 2007; Peng et al., 2007; Misra et al., 2014; Zou et al., 2015). Vsx1 is never co-detected with the V2a or V2b specific markers Chx10 or Gata3 although some Vsx1^+^ cells very likely differentiate into V2a interneurons as they contain Dll4 (Zou et al., 2015). This indicates a sharp downregulation of *Vsx1* expression at the onset of V2 diversification. Interestingly, *Chx10* expression is repressed by Vsx1 in Type 7 cone bipolar cells (Shi et al., 2011) and *Vsx1* expression in retinal cells is inhibited by Chx10 (Clark et al., 2008). Such a cross-repression loop may also exist between these two Prd-L:CVC factors during V2a differentiation and may contribute to rapidly inhibiting *Vsx1* expression in differentiating V2a cells. However, Chx10 distribution was not expanded in the absence of Vsx1, indicating that the latter is not required to restrict *Chx10* expression to the differentiating V2a interneurons. In contrast, lack of co-detection of Vsx1 and Gata3 suggests either that other mechanisms downregulate *Vsx1* expression at the onset of V2b differentiation or that V2b interneurons do not derive from Vsx1^+^ cells. The latter possibility is unlikely since we detected *Vsx1* in the cells expressing *Foxn4* and V2b interneurons derive from *Foxn4*-positive precursors (Del Barrio et al., 2007; Li et al., 2010). Hence, the mechanisms that sharply downregulate *Vsx1* expression at the onset of V2 diversification and the necessity of this tight regulation require further investigations.

Our observations strongly suggest that Vsx1 defines an early intermediate V2 compartment that generates multiple V2 subsets. Whether Vsx1^+^ cells constitute only precursors of the known V2 populations or additionally produce other V2 subsets (Figure 7) remains an open question. The existence of an additional V2 population that derives from *Foxn4*-expressing cells has been demonstrated (Li et al., 2010), and *Vsx1* and *Foxn4* are extensively coexpressed in the early V2 precursors. Therefore, spinal Vsx1^+^ cells very likely produce yet unknown V2 subsets.

Our study of the developmental determinants of the Vsx1^+^ cells identified a novel role for Pax6 in the developing spinal cord. Pax6 is initially present in all progenitor domains of the neural tube and, following neural patterning, is excluded from the p3 domain (Ericson et al., 1997). It is also transiently maintained in V1 (Stam et al., 2012) and in Vsx1^+^ cells (this study). It is necessary for the production of V1 interneurons and slightly impacts on the generation of V2a cells but not of the adjacent V0 or V2b populations (Burrill et al., 1997; Ericson et al., 1997; Sapir et al., 2004; Gosgnach et al., 2006; Zhang et al., 2014). Here we provide evidence that Pax6 is additionally necessary for the expression of *Vsx1* in an early intermediate V2 compartment. The loss of *Vsx1* expression in the spinal cord of *Sey* mutants is unlikely to result from defective dorsoventral patterning or altered neurogenesis in the p2 domain since V2b cells are generated properly and V2a interneurons are only slightly decreased (Burrill et al., 1997; Ericson et al., 1997; Sapir et al., 2004; Gosgnach et al., 2006; Zhang et al., 2014). It more likely manifests a requirement of Pax6 for the expression of *Vsx1* in this early V2 compartment. Identification of other specific markers for these cells will be required to test this hypothesis. In the retina, Pax6 and Vsx1 are present in different cellular compartments (de Melo et al., 2003), suggesting that Vsx1 in the spinal cord may have been recruited into genetic networks different from those that operate in the eye. Consistently, *Vsx1* expression is reactivated after spinal cord injury in the adult zebrafish and a large majority of the Vsx1^+^ cells observed after lesion also contains Pax6 (Kuscha et al., 2012).

In the zebrafish embryonic spinal cord, Vsx1 is detected in common V2a/V2b progenitors and is transiently maintained in part of the V2a lineage. The onset of *Vsx1* expression seems to occur in committed intermediate progenitors, i.e., cells that will undergo a last division to produce pairs of neurons between which Notch signaling will be activated to segregate V2a and V2b interneurons. However, these studies did not exclude the existence of an additional V2 population wherein Vsx1 would be individually present (Batista et al., 2008; Kimura et al., 2008). Compared to our data, these observations suggest that *Vsx1* is initially expressed in a pattern reminiscent of its paralog Chx10, although with an earlier onset, and has been recruited during vertebrate evolution to earlier multipotent neuronal precursors able to generate a large array of V2 subsets. Increased neuronal production and diversification in this early intermediate precursor compartment may have provided motor circuits with specific properties necessary for terrestrial tetrapod locomotion. The detection of murine Vsx1 in this early compartment and the presence in these cells of several markers also found in V2a interneurons are consistent with this late evolution. Functional studies will enable to determine the roles of their derivative populations in the spinal motor circuits and to assess whether they contribute to the execution of original motor capacities present in higher vertebrates.

## Funding

This work was supported by grants from the “Fonds spéciaux de recherche” (FSR) of the Université catholique de Louvain including a FSR-COFUND (Marie Curie) to MH-F, by a “Projet de recherche (PDR)” #T.0117.13 and an “Equipement (EQP)” funding #U.N027.14 of the Fonds de la Recherche Scientifique (F.R.S.-FNRS), by the “Actions de Recherche Concertées (ARC)” #10/15-026 of the “Direction générale de l’Enseignement non obligatoire et de la Recherche scientifique—Direction de la Recherche scientifique—Communauté française de Belgique” and granted by the “Académie universitaire “Louvain””, and by the “Association Belge contre les Maladies neuro-Musculaires asbl” (ABMM) to FC; by the National Institutes of Health (EY020849) to MX; by the Canadian Institutes for Health Research to RLC; and by the Institut National de la Santé et de la Recherche Médicale Grant R05245DS to CP. KM was supported by a grant from the New Jersey Commission on Spinal Cord Research, and HH was funded by the Neuropôle de Recherche Francilien/Région de l’Ile-de-France and by the Fondation pour l’Aide à la Recherche sur la Sclerose En Plaque. MH-F was a Postdoctoral Researcher and FC is a Research Associate of the F.R.S.-FNRS, respectively. SD and AH hold a specialization grant from the F.R.I.A. (Belgium).

## Author Contributions

CF, MH-F, SD, BP and FC designed the experiments. All authors participated in performing the experiments and discussed the data. CF, MH-F, BP, SD and FC contributed to drafting the manuscript.

## Conflict of Interest Statement

The authors declare that the research was conducted in the absence of any commercial or financial relationships that could be construed as a potential conflict of interest.
